# Apoptosis assays with lymphoma cell lines: problems and pitfalls

**DOI:** 10.1038/sj.bjc.6603663

**Published:** 2007-03-06

**Authors:** M J Mattes

**Affiliations:** 1 Garden State Cancer Center at the Center for Molecular Medicine and Immunology, 520 Belleville Avenue, Belleville, NJ 07109, USA

**Keywords:** apoptosis, necrosis, death mechanisms, annexin V, mitochondrial membrane potential

## Abstract

Much attention has been focused on the manner in which tumour cells die after treatment with cytotoxic agents. The basic question is whether cells die via apoptosis or via direct damage from the toxic agent. Various assays have been used to make this distinction. However, we show herein that some of the widely used assays for apoptosis do not in fact distinguish between apoptosis and other forms of cell death. More specifically: (1) A sub-G_1_ DNA content, identified by propidium iodide staining, does not distinguish between apoptotic and necrotic cells; (2) loss of mitochondrial membrane potential does not distinguish between apoptotic and necrotic cells, unless combined with an assay for an intact cell membrane; (3) subcellular fragments that arise from dead cells or from apoptotic bodies can interfere with some assays for apoptosis such as annexin V staining, as they may be close to the size of intact cells, making it difficult to decide where to set the size threshold; (4) irradiated cells display a large increase in nonspecific Ab binding. This may be partly due to an increase in cell size, but, regardless of the cause, it can lead to a mistaken conclusion that there is an increase in a particular antigen if appropriate control reagents are not tested; and (5) experiments utilising Ab crosslinking have neglected the role of cell aggregation, which can cause multiple problems including death from mechanical stress when cells are handled. Consideration of these factors will improve our ability to determine the mode of cell death.

In the continuing attempt to destroy tumour cells while preserving the life of the animal, it is important to understand the mechanism of cell death that is induced by various toxic agents. In recent years, the idea that tumour cells treated with cytotoxic drugs or radiation die owing to apoptosis (programmed cell death), as opposed to the direct effect of the treatment itself, has become widely accepted (Barry *et al*, 1990; Reed *et al*, 1994; Aldridge and Radford, 1998; Ferreira *et al*, 2002; Jazirehi *et al*, 2003; Okada and Mak, 2004). There are, however, a few persuasive skeptics (Brown and Wouters, 1999; Steel, 2001; Gerl and Vaux, 2005). We have begun to investigate the mechanism of cell death induced by radiolabelled antibodies (Abs), primarily using radionuclides emitting low-energy electrons (some Auger and conversion electrons) (Griffiths *et al*, 1999; Ong *et al*, 2001). Unexpectedly, we found that some of the assays widely used to detect apoptosis do not distinguish between apoptosis and other forms of cell death. The purpose of this paper is to describe five of the problems that have been identified. As apoptosis consists of a cascade of biochemical steps, any of these steps can be monitored as a measure of apoptosis. A large number of such assays have been used and most of them are reliable. However, (1) cells having sub-G_1_ DNA content have been assumed to be apoptotic (Chan *et al*, 2003; Jazirehi *et al*, 2003), but various types of cell fragments also may have a sub-G_1_ DNA content, and we demonstrate herein that three types of necrotic cell preparations have large numbers of cells with sub-G_1_ DNA; (2) similarly, although apoptotic cells lose mitochondrial membrane potential (Darzynkiewicz *et al*, 2001) and although the mitochondrial membrane is a major focus of apoptotic pathways (Green, 2003; Henry-Mowatt *et al*, 2004), we show that necrotic cells also lose mitochondrial membrane potential; (3) subcellular fragments can potentially interfere with assays such as annexin V staining. Apoptosis and other types of cell death usually generate subcellular fragments, and these may be mistaken for apoptotic cells; (4) cell damage owing to irradiation causes an increase in nonspecific Ab binding (to viable, non-apoptotic cells), which can lead to false conclusions regarding Ab specificity if negative control Abs are not tested; and (5) Ab binding can induce tight aggregation of cells, especially if a secondary Ab is included. Such aggregates are sometimes impossible to disperse by the usual methods, without killing the cells, and therefore these cells cannot be evaluated for apoptosis, or any other property, by many of the commonly used methods.

These results are not meant to imply that apoptosis is not the mode of cell death in many situations, for both normal and tumour cells. Some new anticancer drugs are specifically designed to activate apoptosis, and probably act via this mechanism (Gerl and Vaux, 2005). However, in regard to classical cytotoxic drugs and radiation, some re-evaluation of the literature, based on the factors described herein, may be required in order to correctly understand the mechanism of cell death.

## MATERIALS AND METHODS

### Cell lines, Abs, and other reagents

The Jurkat T-lymphoma and Ramos and Raji B-lymphoma cell lines were obtained from the American Type Culture Collection (Manassas, VA, USA). They were grown in RPMI 1640 medium containing 12.5% foetal bovine serum and supplemented with glutamine and penicillin/streptomycin (Invitrogen, Grand, Island, NY, USA). Cells were tested routinely for mycoplasma by the Mycotect assay (Invitrogen) and were negative. Antibody L243 is an IgG2a Ab to HLA-DR, and was produced by the hybridoma obtained from ATCC, as described previously (Ong *et al*, 2001). Annexin V conjugated to Alexa 488 was from Molecular Probes (Eugene, OR, USA). Digitonin was from Sigma Chemicals (St Louis, MO, USA, #37006) and other drugs and chemicals were also from Sigma.

### Induction of necrosis or apoptosis

Stock etoposide (Sigma Chemicals, #E-1383,=VP-16) was 100 mM in DMSO (dimethylsulfoxide) and was stored at −20°C. It was used at a final concentration of 30 *μ*M (Amarante-Mendes *et al*, 1998), and was incubated with cells for 16–18 h under standard tissue culture conditions. Stock camptothecin (CTT, Sigma #C-9911) was 10 mM in DMSO, also stored at −20°C. It was used at a final concentration of 4 *μ*M (Johnson *et al*, 1997), and was incubated with cells for 4 h. Stock paclitaxel (the pharmaceutical from Mead Johnson, Princeton, NJ, USA) was at 6 mg ml^−1^, as supplied, and stored at room temperature. It was used at a final concentration of 40 nM (Jazirehi *et al*, 2003) and was incubated with cells for 18–20 h. For the drugs dissolved initially in DMSO, DMSO alone at the same concentration was tested as a control: this always had very little or no effect. Heat treatment was at 56°C for 45 min, which was the minimum time required to kill all of the cells, as assayed by trypan blue exclusion. Cells were heated in tissue culture medium, then diluted and plated under normal tissue culture conditions. Treatment with digitonin was at 0.005% for 5 min. The digitonin was dissolved by heating at 100°C for 3 min in water at a concentration of 1.0% just before use. It was diluted 1 : 5 in phosphate-buffered saline (PBS), then diluted 1 : 40 to the cell suspension, which was in sterile Dulbecco's phosphate-buffered saline (DPBS) (Invitrogen), 0.5% bovine albumin, and 10 mM NaN_3_. To kill cells by freeze–thawing, cells in 1.0 ml tissue culture medium were snap-frozen in 2-methyl-butane, cooled with dry ice and then thawed at 37°C. This killed more than 90% of the cells. Cells were irradiated with a ^137^Cs irradiator (JL Sheppard, San Fernando, CA, USA) at the University of Medicine and Dentistry of New Jersey (Newark, NJ, USA). The time required to deliver 10 Gy was approximately 7 min.

### PI staining of DNA content

Cells were washed once with tissue culture medium without phenol red and then suspended in 0.5 ml of 0.1% sodium citrate (3.4 mM) and 0.1% Triton X-100 containing 50 *μ*g ml^−1^ propidium iodide (PI) (Nicoletti *et al*, 1991). Analysis was with a FACSCalibur (BD Biosciences, San Jose, CA, USA).

### JC-1 staining of mitochondria

JC-1 (Sigma) was dissolved at 1.0% in DMSO and stored at −20°C. A 1/100 volume was added to approximately 5 × 10^5^ cells in approximately 130 *μ*l of tissue culture medium. After 10 min at 37°C, cells were washed twice with tissue culture medium, the second time without phenol red, then suspended in 0.5 ml for fluorescence-activated cell sorter (FACS) analysis. This assay produced results identical to the Mitoscreen assay of BD Biosciences (San Diego, CA, USA). The nuclear stain Sytox green was obtained from Molecular Probes at 5 mM in DMSO and stored at −20°C. The stock was prepared by diluting 2 *μ*l into 1.0 ml tissue culture medium without phenol red, and 1/100 volume of this was added to the cell suspension. After 5 min at room temperature, samples were analyzed on a FACSCalibur and were also examined on an Olympus BH2 fluorescent microscope with a 100 W mercury bulb.

### Annexin V staining

Cells were washed once with tissue culture medium without phenol red supplemented with 2 mM CaCl_2_. After discarding the supernatant, 5 *μ*l of the annexin V conjugate was added to the residual volume (approximately 130 *μ*l). After 45 min at room temperature, cells were washed twice, then suspended in the same medium containing 1 *μ*g ml^−1^ PI.

### Immunofluorescent staining

Cells were stained either with or without fixation and permeabilisation. Unfixed cells were stained in DPBS (Invitrogen), 0.5% bovine albumin, and 10 mM NaN_3_. Fixed and permeabilised cells were stained following methods developed to detect cleaved poly(ADP-ribose) polymerase (PARP) (an intracellular antigen), although in this study we show only results of nonspecific staining. Cells were fixed with 4% formaldehyde (prepared from depolymerised paraformaldehype; Farr and Nakane, 1981) in PBS with 0.15% saponin (Sigma Chemicals) for 20 min at 4°C. All buffers used subsequently also contained 0.15% saponin. After washing, the pelleted cells were suspended in the residual volume in the tube (approximately 130 *μ*l) and 10 *μ*g of the Ab was added in a small volume (<5 *μ*l). The Abs used were either FITC-goat anti-mouse IgG (Cappell Laboratories, now ICN, Irvine, CA, USA) or FITC-normal goat IgG F(ab′)_2_ fragment (Jackson ImmunoResearch, West Grove, PA, USA). Cells were incubated for 30 min usually at room temperature, washed twice, then examined on a FACSCalibur and a fluorescent microscope.

### Analysis of Ab-induced cell aggregation

Abs were added at the indicated concentrations and cells were then incubated under tissue culture conditions in a 24-well plate. The standard method of harvesting the cells, which caused dispersal of loose aggregates, was to repipette eight times with a short (6 inches) Pasteur pipette. To remove unbound Ab after 24 h, from cells aggregated by L243 without damaging the cells, the cells were gently transferred to a 15 ml tube using a truncated pipettor tip (with a large opening). The cell aggregates were spun gently (1 min, 400 × G) and washed four times with 12 ml of tissue culture medium. They were then transferred to a T25 flask containing 20 ml medium and incubated for 10 days until they had multiplied 16-fold. Accurate cell counts of aggregated cells could not be obtained until the aggregates had dispersed, which required approximately 8 days. The fraction surviving was calculated from the growth curves as described previously (Griffiths *et al*, 1999). Work with 1F5 generally followed Shan *et al* (1998). Cells (2.5 × 10^5^) were incubated in a 24-well plate in 0.5 ml of tissue culture medium containing 1F5 at 10 *μ*g ml^−1^ for 20 min at 37°C, then a secondary Ab, goat anti-mouse IgG Fc*γ*, F(ab′)_2_ fragment (Jackson ImmunoResearch) was added to a final concentration of 50 *μ*g ml^−1^, and the incubation continued overnight.

## RESULTS

### Sub-G_1_ DNA content, assayed by staining with PI

**Figure 1:**
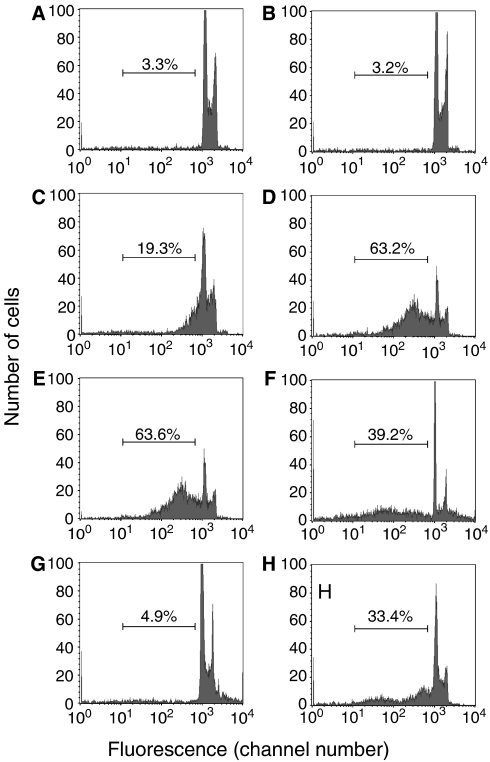
Necrotic cells can have sub-G_1_ DNA content. Histograms of cells stained with propidium iodide, a DNA stain, are shown. (**A**) Control Jurkat cells. (**B–E**) Jurkat cells were killed by heating at 56°C and analyzed immediately after killing (**B**), or after incubation at 37°C for 24 h (**C**) or 48 h (**D**). (**E**) is the same as (**D**), except with a higher threshold setting, which excludes all cells smaller than the smallest normal cell. (**F**) Jurkat cells were killed by treatment with 0.005% digitonin for 5 min, then washed, and incubated for 6 h at room temperature. (**G**, **H**) Jurkat cells were killed by freeze–thawing and analyzed immediately after killing (**G**), or after incubation at 37°C for 3 days (**H**). The bar shows the region considered to represent cells with sub-G_1_ DNA content and the percentage of cells within this region is shown.

Necrotic cells were produced by two methods that have been used by others for this purpose: treatment with heat (Rubartelli *et al*, 1997) or the mild detergent digitonin (Single *et al*, 1998). They were also produced by freeze–thawing. In all three cases, a sub-G_1_ DNA peak was seen corresponding to fragmented cells (Figure 1). This effect was not observed immediately after heat treatment or freeze–thawing even though the cells were virtually all dead at this time, as assessed by trypan blue staining (Figure 1B and G). But when these killed cells were incubated for 1–3 days at 37°C, a large sub-G_1_ fraction appeared (Figure 1C, D and H), which can be attributed to gradual disintegration of the dead cells. Freeze–thawing caused some aggregation of the cells at time 0 (Figure 1G), but these aggregates appeared to disperse by the later time points (Figure 1H). Morphologically, after trypan blue staining, freeze–thawed cells showed gradual disintegration, but there were no evident changes in the heat-killed cells. These results are potentially dependent on the setting of the threshold, which is the cutoff that separates cells from subcellular debris, based on the size of the particles. This is a major issue in assays of apoptosis or other types of cell death, as dead or apoptotic cells commonly generate subcellular fragments and a single cell can generate multiple fragments (see the section on annexin V staining below.) As apoptotic cells are sometimes condensed relative to normal cells, the threshold cannot be set based on the size of normal cells. In most of the data shown, we used a threshold of approximately 1/2 the size of the smallest normal (untreated) cell, which was chosen to reduce the possibility that a single cell could generate multiple fragments that would be counted as cells. However, this is not entirely satisfactory, as normal cells vary in size by approximately twofold (in terms of forward scatter on the FACS). To assess the impact of this factor, we determined how much the results would be affected by different threshold settings. Figure 1E shows that with a threshold set to the size of the smallest normal cells, which is the highest reasonable setting, there was still a large sub-G_1_ fraction in the heat-killed cells that had been incubated for 2 days, and similar results were obtained with the freeze–thawed cells.

With cells killed by digitonin, also (another way of inducing necrosis), a large sub-G_1_ fraction was present (Figure 1F). After a 5 min incubation with 0.005% digitonin, virtually all of the cells were lysed, as determined by trypan blue staining. When assayed for DNA content, a sub-G_1_ DNA peak was evident immediately after digitonin treatment: at time 0, the percentage of cells with a sub-G_1_ DNA content was 24.5%. This value increased during a 6 h incubation in tissue culture medium at 37°C to 39.2% (Figure 1F shows results at the 6 h time point). We conclude that necrotic cells can have a sub-G_1_ DNA content and therefore, this assay cannot distinguish between apoptotic and necrotic cells.

### Mitochondrial membrane potential assayed by JC-1 staining

**Figure 2:**
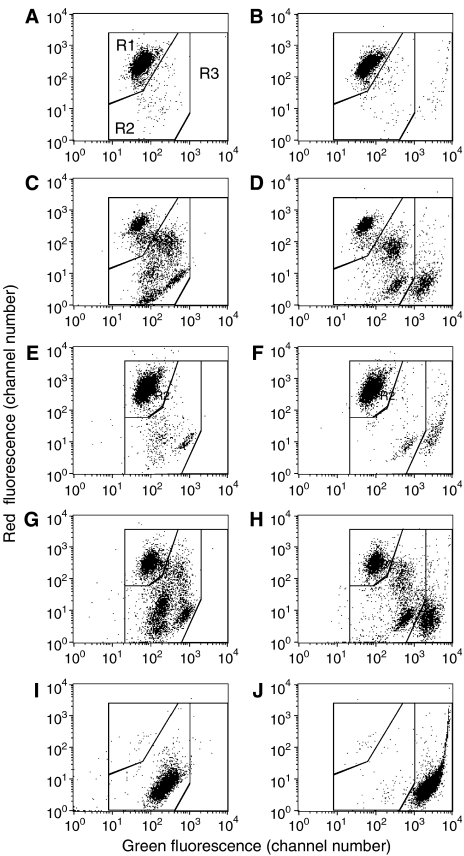
Necrotic cells lose mitochondrial membrane potential (MMP), assayed by JC-1 staining, but can be distinguished from apoptotic cells by inclusion of Sytox green. Jurkat or Ramos cells were stained with JC-1 only (left graphs) or with JC-1 plus Sytox green (right graphs), which is a nuclear stain selective for dead cells. (**A**, **B**) Untreated Jurkat cells. (**C**, **D**) Jurkat cells treated overnight with 100 *μ*M etoposide. Note that the apoptotic cells include two distinct populations, which can be considered early and late apoptotic cells based on the gradual loss of red staining. (**E**, **F**) Untreated Ramos cells. (**G**, **H**) Ramos cells treated with 20 nM paclitaxel overnight. (**I**, **J**) Jurkat cells killed by heating at 56°C for 45 min and examined immediately after killing. Regions R1, R2, and R3 indicated in (**A**) represent the normal, apoptotic, and lysed cells, respectively, after staining with JC-1 plus Sytox green. Note that these regions are slightly different, but similar for the two cells lines.

Jurkat cells lysed by heating at 56°C lost all bright red mitochondria staining from JC-1, as observed by FACS (Figure 2I) and by microscope. Similar results were obtained with cells killed by freeze–thawing (data not shown). Control cells had the expected bright red mitochondrial staining (Figure 2A). Therefore, these necrotic cells would be mistakenly considered apoptotic, according to the standard JC-1 staining protocol. We conclude that this assay also does not distinguish between apoptotic and necrotic cells. We attempted to distinguish between lysed and apoptotic cells by adding a nuclear stain, Sytox green, for dead cells. Although JC-1 also produces green staining in regions in which it is less concentrated than in healthy mitochondria, the staining of dead cells by Sytox green was much brighter at the concentrations used. To demonstrate the value of combining Sytox green with JC-1 staining, we examined Jurkat cells treated with 100 *μ*M etoposide, a standard model for apoptosis. At the time point examined, 18 h, both apoptotic and lysed cells were present. By visible light microscopy, there were approximately 25% dead cells (stained with trypan blue) and 30% apoptotic cells (unstained with trypan blue and abnormal morphologically, many having a condensed, fragmented nucleus). As shown in Figure 2C, JC-1 staining alone did not distinguish between apoptotic and necrotic cells, both of which had reduced red staining and would therefore be considered to be in the ‘apoptotic’ region of the dot plot. But when Sytox green was included, lysed and apoptotic cells were clearly distinguished (Figure 2D). The apoptotic cells consisted of two distinct populations, which can be considered early and late apoptotic cells, based on their loss of red fluorescence, and cells intermediate between these two populations were also evident. In this context, we refer to ‘late’ apoptotic cells as those which have lost essentially all red mitochondrial staining by JC-1, but still have an intact plasma membrane, so are not stained by Sytox green. Note that apoptotic cells had brighter green staining than normal cells, as well as diminished red staining, and that the green staining was entirely due to JC-1, not Sytox green, as shown by comparing Figure 2C and D. Similar results were obtained with another apoptosis model, Ramos B-lymphoma cells treated overnight with 40 nM paclitaxel (Figure 2G and H). Application of the Sytox green method to heat-killed Jurkat cells demonstrated that all of the cells were in the ‘lysed cell’ region of the dot plot (Figure 2J), as expected as all of the cells stained with trypan blue. Thus, this method correctly identified lysed cells, and distinguished them from apoptotic cells. In conclusion, necrotic cells would be misidentified as apoptotic cells by the conventional JC-1 assay, but are correctly characterised if Sytox green is included.

### Annexin V staining and the problem of subcellular fragments

**Figure 3:**
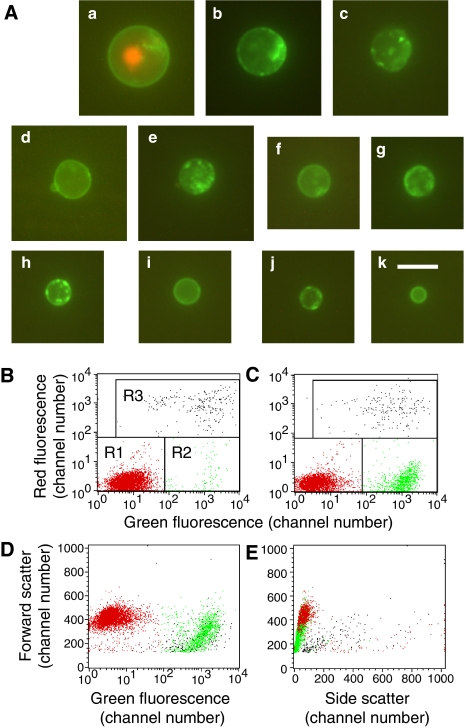
Annexin V-positive, propidium iodide (PI)- negative cells, and subcellular fragments after 4 h treatment of Jurkat cells with 4 *μ*M camptothecin. (**A**) Representative photographs showing ‘apoptotic cells’ of varying sizes and a lysed cell (a) stained with both annexin V and PI for comparison. The ‘apoptotic cells’ range in size from 12.2 *μ*M (b) to 3.8 *μ*M (k). The diameter of normal Jurkat cells is 11.0±1.0 *μ*M, so slightly smaller than the cell shown in (b). Bar=10.0 *μ*M. FACS dot plots show control cells (**B**) or CTT-treated cells (**C**) stained with annexin V (green) and propidium iodide (red). The marked regions R1, R2, and R3 include healthy, apoptotic, and lysed cells, respectively, and apoptotic cells are coloured green, normal cells red, and lysed cells black. (**D**) The same cells as in (**C**), with the same colouring, are shown in a plot of forward scatter vs. green fluorescence. Note that the ‘apoptotic cells’ (green) are smaller then normal cells (red), and of varying size, so there is no obvious separation between apoptotic cells and subcellular fragments. (**E**) Similar to (**D**), but a plot of forward scatter *vs* side scatter, further showing the overlap between normal cells, apoptotic cells, and subcellular fragments.

Unlike the two previous methods, staining with Alexa 488 annexin V together with PI gave excellent differentiation between lysed cells, apoptotic cells, and normal cells, when applied to all of the apoptotic or heat-killed populations described above. Annexin V reacts with phospholipids that are normally on the inner face of the cell membrane, but move to the outer face in apoptotic or necrotic cells (Ran *et al*, 2005). Like the modified JC-1 assay, this assay identifies lysed cells by using a dead cell-specific nuclear stain, in this case PI. However, a significant problem with the assay is the presence of green (annexin-stained) subcellular fragments. Small subcellular material is routinely gated out in FACS analysis by size (measured by forward scatter), but the problem is that the ‘apoptotic cells’ (annexin V positive, PI negative) formed a continuum, ranging from the size of normal cells to small vesicles that are clearly subcellular, with no natural separation between the two. This is shown in Figure 3, with Jurkat cells treated with 4 *μ*M CTT for 4 h. Figure 3A shows representative examples of cells or cell fragments of various sizes. The annexin V-stained objects ranged in diameter from the size of normal Jurkat cells (11.0±1.0 *μ*M) to 3.8 *μ*M. Some were stained in a patchy pattern and others had very homogeneous membrane staining, as shown. There was a tendency for smaller objects to more commonly have the homogeneous staining pattern, although the staining pattern was not strictly correlated with the size of the objects. Figures B and C show the typical display of apoptotic cells using a dot plot of FL2 (red) *vs* FL1 (green). Although the induction of apoptotic cells by CTT is clear, the calculation of the % apoptotic cells strongly depends on the size of the objects that are included in the count. To illustrate this problem, Figure D shows forward scatter *vs* FL1 for the same cells using the colour scheme from Figure 3C. These results demonstrate that ‘apoptotic cells’ are smaller than normal cells, and that many are sufficiently small that they must be considered subcellular. Figure 3E shows a similar plot of forward scatter *vs* side scatter, further demonstrating the relationship between normal cells and ‘apoptotic cells’. We generally use a cutoff equal to half the size of the smallest healthy cell (as judged by the forward scatter, which is proportional to cell size). With this cutoff (at a value of 145) the % apoptosis was calculated to be 21.6%. However, if the cutoff used was just below the size of the smallest normal cell (at a value of 290), which is also reasonable, then the calculated % apoptosis would be only 13.8%, which represents a reduction of 36%. As the cutoff size is arbitrary, the calculated % apoptosis is also arbitrary. These data do demonstrate the induction of apoptosis by CTT, as some of the annexin V-positive, PI-negative objects were cell size, but there is considerable uncertainty regarding the % apoptotic cells, as normally calculated.

### Staining for cleaved PARP: nonspecific staining

**Figure 4:**
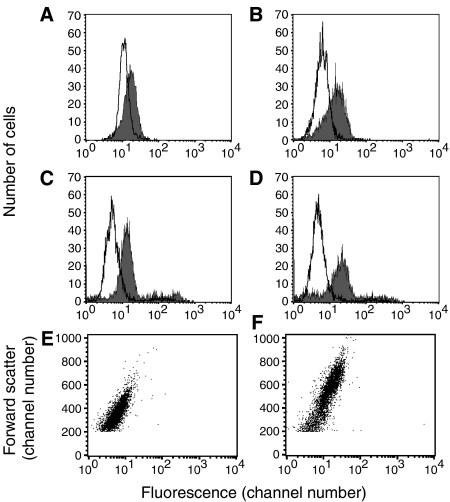
Nonspecific staining of irradiated cells with fluorochrome-conjugated secondary Abs. Raji cells were irradiated with 10 Gy from a ^137^Cs irradiator, then washed and cultured in tissue culture medium for 1–3 days. At day 1 (**A**) or day 2 (**B**), cells were fixed and permeabilised, then stained by the procedure used for staining with anti-cleaved PARP (but without the primary Ab), using FITC-goat anti-mouse IgG. At day 2 (**C**) or day 3 (**D**) viable unfixed cells were stained with FITC-normal goat IgG. Results are shown for untreated cells (unfilled curve) and irradiated cells (filled curve). (**E**, **F**) Dot plots of forward scatter *vs* green fluorescence for the same two samples shown in (**B**): (**E**), control untreated cells; (**F**), cells 2 days after irradiation. Note that the irradiated cells are considerably larger and that fluorescence is correlated with cell size (as indicated by forward scatter).

In investigating the cleavage of PARP by immunofluorescence, we noted that irradiated but not control cells had quite strong nonspecific staining with the second Ab only (FITC-goat anti-mouse IgG). Representative results are shown in Figure 4A and B. This staining was seen at day 1 after irradiation with 10 Gy and became increasingly stronger on days 2 and 3. Radiation delivered via Abs binding to the cell surface in the form of ^111^In-rituximab produced very similar results (data not shown). Results are shown with Raji B lymphoma cells, but similar results were obtained with another B lymphoma cell line, Daudi. Similar staining was observed with 13 different Abs, all expected to be control, non-reactive Abs, and all non-reactive with untreated cells. The panel of Abs tested included conjugates with fluorescein, rhodamine, Alexa 488, and phycoerythrin, and included goat and mouse Abs. Five of these were F(ab′)_2_ fragments, demonstrating that the Fc receptor was not involved, and some of the Abs (from Jackson ImmunoResearch) had been absorbed with human serum proteins. The initial experiments utilised fixed and permeabilised cells, but the same experiments were then performed with unfixed cells to investigate cell surface staining. Results with the unfixed cells were essentially the same as with the fixed and permeabilised cells (Figure 4C and D), indicating that the staining was of the cell surface. Staining was virtually identical whether the Ab incubation was at room temperature or 4°C, demonstrating that cell metabolism was not required for this staining to occur. The radiation dose used in these experiments kills approximately 99.9% of the cells, but at these relatively early time points, out to 3 days, the cells were essentially 100% viable, although enlarged, and there were no morphological abnormalities observed by phase contrast (ie, the cells did not look apoptotic morphologically). In addition, these cells did not stain with annexin V and had bright red mitochondria after staining with JC-1, further demonstrating that they were not apoptotic. The significance of these results for our present purpose is that this staining could be misinterpreted as specific staining of cleaved PARP, cleaved caspase 3, or whatever other antigen was targeted, if non-reactive control Abs were not tested. Although it can be argued (and it is true) that such control Abs should always be tested, in reality they are sometimes omitted, as discussed further below. The explanation for this nonspecific staining of irradiated cells is under investigation. It seems likely to be related in part to the increase in the size of the cells after irradiation. Figure 4E and F show a plot of forward scatter *vs* green fluorescence for control cells (C) or irradiated cells (D). The irradiated cells are considerably larger (as indicated by forward scatter) and for both samples, the larger cells have more fluorescence than the smaller cells. By comparing these two graphs, it is evident that the level of nonspecific fluorescence is primarily dependent on the size of the cell. In conclusion, irradiated cells have much higher nonspecific Ab binding than control cells, which means that untreated cells cannot be used as a specificity control in experiments of this type.

### Cell aggregation by Abs

**Figure 5:**
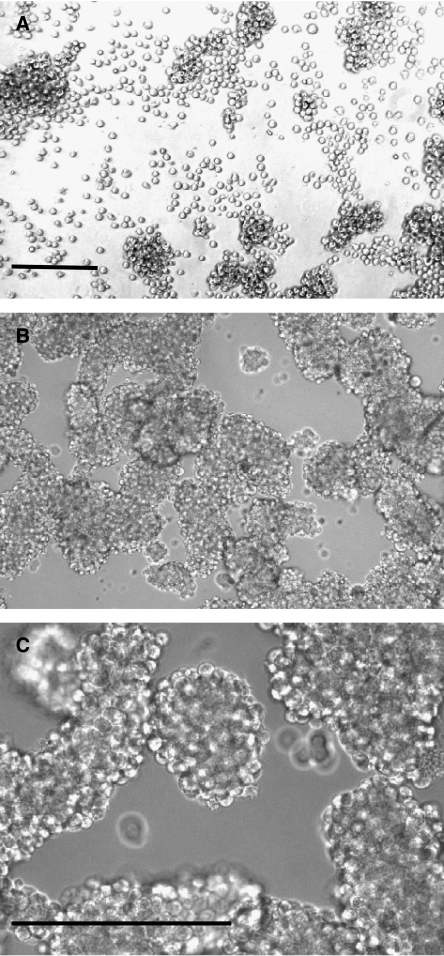
Tight aggregation of Raji B lymphoma cells by anti-HLA-DR (L243). (**A**) Control cells, which form some loose clusters that can be readily dispersed by pipetting (without loss of viability). Note that there are also many single cells (× 10 objective, phase contrast). (**B**) Cells incubated overnight with L243 at 30 *μ*g ml^−1^. The cells form large, irregular clusters and there are very few single cells. The cell density seems greater than in (**A**) because all of the cells in the well are clustered into the centre of the well (× 10 objective, brightfield). (**C**) A higher-power photograph of the same cells as shown in (**B**) to better display the tightly aggregated cells (× 40 objective, brightfield). Bar= 200 *μ*M.

Binding of Abs to the cells surface has been frequently reported to induce apoptosis of lymphoma cells, especially if the Ab is crosslinked with a secondary Ab (Truman *et al*, 1997; Shan *et al*, 1998; Nagy *et al*, 2002; Carlo-Stella *et al*, 2006). In performing such experiments, we noted that Ab binding sometimes induced extensive aggregation of the cells. This is shown for Raji cells treated with L243 (anti-HLA-DR) at 30 *μ*g ml^−1^ (Figure 5). These aggregates were very large and very tight, such that it was impossible to disperse the cells into a single-cell suspension. That is, shaking or repipetting the aggregated cells only partially dispersed the aggregates, leaving clusters of 10 cells or more. Very hard repipetting did gradually disperse the aggregates, but appeared to kill many of the cells, especially those that were released as single cells, as detected by trypan blue staining. The remaining aggregates often had dead cells at the edges of the aggregates, while otherwise the aggregates remained mostly viable. These results suggested that the mechanical trauma required to extract cells from the clusters was responsible for the death of the cells. Because of the large aggregates, it was impossible to count the cells with an acceptable level of accuracy, and it was also impossible to test the cells for apoptosis by most of the methods that are usually employed. The aggregates started to develop within 2 h and became increasingly large and dense over the next 24 h. Initial experiments utilised L243 at 30 *μ*g ml^−1^, following Nagy *et al* (2002), but a range of Ab concentrations were tested. Judging microscopically by the extent of aggregation, 3 *μ*g ml^−1^ had the same effect as 30 *μ*g ml^−1^, 30 ng ml^−1^ produced slight aggregation, and 300 ng ml^−1^ had an intermediate effect.

One method of evaluating the viability of cells within clusters is a long-term proliferation assay (Griffiths *et al*, 1999), which simply consists of cell counts at various times, out to 21 days. With this assay, the cells are cultured until the clusters disperse, due presumably to the gradual catabolism of the bound Ab. Unbound Ab was removed after 1 day by washing the cells, which could be performed (without damaging the cells) by transferring the aggregates gently with a truncated pipettor tip (having a large opening). Accurate cell counts could not be obtained until clusters became small, which required 8 days; 11 days were required for Ab-induced aggregation to be totally eliminated. Calculation of the apparent fraction surviving from the growth curves suggested that the fraction surviving was only 0.168. However, this calculation is based on the growth rate. As there was no any indication of significant cell death (cells stained with trypan blue or cells that looked apoptotic), the most likely explanation for these data is that the aggregated cells multiplied more slowly than untreated cells, due perhaps to physical constraints. The calculated doubling time was 1.37 days for Ab-treated cell and 0.83 days for control cells. Although the explanation for the decreased growth rate is uncertain, it is reasonable to conclude that a decreased growth rate does not necessarily reflect either apoptosis or any other type of cell death.

The aggregation of cells by Abs and the tightness of the aggregates was dependent on the particular cell line as well as on the Abs used. RL cells were aggregated by L243, but did not develop the extremely tight undissociable aggregates that formed with Raji cells. The anti-CD20 Ab 1F5 was also tested, alone or with a secondary Ab, because this is a model that has been used by other laboratories. Using the model described by Shan *et al* (1998), Raji cells incubated with 1F5 plus a secondary Ab (but not with 1F5 alone) developed tight aggregates that could not be entirely dispersed by standard repipetting, and harsh pipetting appeared to cause significant levels of cell death. These clusters, however, were much smaller and less tight than the clusters induced by L243. With other cell lines, namely Ramos, Daudi, RL, and SU-DHL-6, under the same conditions, there was some Ab-induced clustering, but dispersal of the clusters produced a high-viability single-cell suspension with no increase in the number of dead cells. Thus, based on the limited studies that have been performed, it appears that Raji is more susceptible than some other cell lines to tight Ab-induced clustering.

## DISCUSSION

The data presented demonstrate five problems with assays that are widely used to measure apoptosis, which might lead to misinterpretation of the results. Table 1 provides a synopsis of the four assays utilised, and their potential problems. The aggregation problem would interfere with all of these assays. Three of these problems might be considered technical, in that they relate to the quality of the experiments performed. The other two, however, relate to the validity of the assays themselves: that is, the assays do not distinguish between apoptotic and necrotic cells. This difference is not critical in some cases, in which the main question is whether cells are dead or viable. In such a case, this problem may be considered mainly semantic, as these two assays do show unequivocally that cells are dead. However, in cases in which the focus is on the mechanism of cell kill, this factor is fundamental. What is demonstrated herein, specifically, is that some cells dead from necrosis may be mistaken for apoptotic cells. Our data relate only to non-adherent haematological tumours and may not entirely apply to adherent tumour cells. However, it seems likely that most of the problems discussed, except for the problem of cell aggregation, would also apply to adherent cell lines.

The PI method of staining sub-G_1_ cells was developed using corticosteroid-treated thymocytes (Nicoletti *et al*, 1991). With this cell type, the assay correlated well with other assays for apoptosis, and cells killed by non-apoptotic mechanisms were negative. However, the cells that had been killed by other mechanisms were not maintained and examined for several days, as performed herein, and may have developed a sub-G_1_ population at later time points. In addition, thymocytes may behave differently from tumour cells in this assay. The use of a 1–2-day incubation period, as performed here, is relevant to most toxicity studies. It is not surprising that necrotic cells gradually disintegrate, slowly losing DNA. It might be argued that in our experiments apoptosis was activated during the 45 min heat treatment (at 56°C), which was the time interval required to lyse all of the cells (assessed by trypan blue staining). However, it is unlikely that apoptosis could proceed at 56°C. Mild heat treatment, with a peak at 43°C, does induce apoptosis in haematological cell lines, but temperatures above 47°C induce only necrosis (O'Neill *et al*, 1998). Also, as freshly heat-killed cells did not have a sub-G_1_ cell population, this hypothesis would require that apoptotic processes continued to function after the cells lysed, which is also unlikely. Moreover, this argument would not apply to cells killed by the other two methods used. Killing with digitonin required only a 5 min incubation to produce a significant population of sub-G_1_ cells. Freeze–thawing killed cells within seconds, if we do not count the time for which the cells were frozen.

The use of assays for MMP to detect apoptosis was based on the finding that apoptotic pathways induce leakage of components from inside the mitochondria, which play a major role in the further amplification of apoptotic pathways (Green, 2003; Henry-Mowatt *et al*, 2004). Although such assays have been widely used (Darzynkiewicz *et al*, 2001; Chan *et al*, 2003; Jazirehi *et al*, 2003; Carlo-Stella *et al*, 2006), there has been insufficient consideration of the fact that necrotic cells also lose their MMP. There were a few previous reports that cells killed by any mechanism have non-functional mitochondria (Darzynkiewicz *et al*, 1994; Lemasters *et al*, 1998). We demonstrated that necrotic cells killed by heating or freeze–thawing lost MMP, demonstrating that this assay, like the PI assay, cannot identify apoptotic cells. However, we also demonstrated that if determination of cell membrane integrity is combined with an assay of mitochondrial function, then it is possible to reliably distinguish between viable, apoptotic and lysed cells. This was demonstrated with two model systems: etoposide-treated Jurkat cells and paclitaxel-treated Ramos cells. Inasmuch as paclitaxel treatment of Ramos cells was reported to be unable to induce apoptosis in the absence of an anti-CD20 Ab (Jazirehi *et al*, 2003), we note that in our studies this was not the case. We have no explanation for this discrepancy, but note that the cell density was much greater in the experiments of Jazirehi *et al* (2003) than in our experiments. The presence of apoptotic cells after treatment of Ramos with paclitaxel was confirmed by microscopic observation (cells with condensed, often fragmented nuclei unstained by trypan blue)by annexin V plus PI staining, and by immunofluorescent staining with an Ab to cleaved PARP (unpublished data). The modified JC-1 assay may be widely applicable, as it is a simple and rapid assay that enumerates apoptotic, lysed, and normal cells.

The other three problems described here relate to the quality of the assays performed rather than the validity of the assays themselves. However, these problems can also lead to overestimation of the role of apoptosis in the killing of cells. Because dead cells gradually disintegrate, producing subcellular fragments of various sizes, because apoptotic cells are sometimes condensed, and because apoptotic cells often disintegrate in the form of apoptotic bodies (Kerr *et al*, 1972), the calculation of percentage apoptosis is complicated by the presence of subcellular fragments. Moreover, we recently showed that small annexin V-positive vesicles are shed from viable cells in the absence of significant levels of apoptosis or cell death (Michel *et al*, 2006). Although subcellular fragments can be gated out by FACS analysis, the problem is where to set the gate, since, as shown here, the subcellular fragments form a continuum of sizes, without any clear distinction between apoptotic cells and subcellular fragments. Our standard cutoff, half the size of the smallest normal cell, seems reasonable, but it should be recognised that it is not entirely satisfactory. As the size of normal cells covers an approximately twofold range, and as damaged cells are frequently enlarged (especially irradiated cells), this cutoff size will be less than one-fourth the size of some viable cells. Therefore, it is possible that one affected cell can fragment into multiple ‘apoptotic cells’, which would distort the percentages calculated. Thus, annexin V-positive, PI-negative objects can be accepted as apoptotic cells only if those objects are shown to be approximately cell-size. As phospholipid asymmetry is maintained by an energy-dependent enzyme (Verhoven *et al*, 1995), it is likely that it would be lost in any type of subcellular fragment. If these fragments do not contain DNA or have an intact membrane, they will not stain with PI and might be recorded as apoptotic cells. In the particular experiment shown, the calculated % apoptosis was altered by 36% by changing the size threshold, which might be considered a relatively minor difference (though not insignificant). But the percentage of annexin V-positive subcellular fragments could be much higher in other cases. Published data should include a graph of forward scatter (as in Figures 3D and E), the size threshold used for forward scatter, or photographs of representative apoptotic cells, so that the size of the annexin-stained objects can be evaluated.

It may seem unnecessary to argue for the importance of testing non-reactive, isotype-matched control Abs, labelled in the same way as the specific Ab, as a test of specificity, but this is not the case. Such controls are sometimes lacking or, at least, not described. This problem is due partly to the fact that some of the Abs widely used to detect apoptosis are rabbit monoclonal Abs, for which the required control reagents are not available. For example, BD Biosciences and Cell Signaling Technology (Beverly, MA, USA) sell fluorescein-conjugated rabbit monoclonal Abs to apoptosis-related antigens, but they do not sell a non-reactive control rabbit Ab conjugated similarly (although they do sell mouse and rat control Abs). Technical information from these suppliers and statements from other investigators (Roederer, 2001) have claimed that isotype-matched control Abs are not required to prove specificity, and are not a reliable control. However, such arguments are unconvincing: if a non-reactive Ab produces the same staining as a specific Ab, then there is no specificity. Inasmuch as there is a single class of rabbit IgG (Knight *et al*, 1985), normal rabbit IgG can be used as a control for a rabbit mAb, but it must be appropriately conjugated. It is sometimes considered adequate to use untreated cells as a specificity control, in comparison with cells treated with an agent that induces apoptosis. However, our data with irradiated cells demonstrate that such a control is not an adequate proof of specificity. Any cell that is damaged may potentially have increased nonspecific binding. Irradiated cells become enlarged and cell enlargement probably explains part or all of the effect observed. We note that PARP cleavage is frequently assayed by Western blotting, which would not be affected by the problem described here, but does not provide information on individual cells.

The problem of cell aggregation by Ab crosslinking is not a problem of the apoptosis assays themselves, but it is relevant to the current study because this is another factor that may lead to overestimation of the role of apoptosis. This factor appears to have been overlooked in some previous reports. For example, earlier studies of the effect of L243 (anti-HLA-DR) on Raji cells (identical to the model we used), which claimed that apoptosis occurred, did not mention the dramatic aggregation that occurs (Kostelny *et al*, 2001; Nagy *et al*, 2002). One consequence of aggregation, which we documented, is that it may be impossible to disperse the cells in tight aggregates without killing the cells. Such death can be attributed to mechanical stress, as the cells are handled (pipetted, centrifuged, or resuspended). Whether damage occurs or not depends on the strength of the crosslinking. Loose aggregates, such as those that form spontaneously with many B-lymphoma and B-lymphoblastoid cell lines, are readily dispersed by pipetting or shaking, with no evident damage to the cells. However, when crosslinking is strong, as when Raji cells are treated with Ab L243, it may be impossible to disaggregate the cells without killing them. Thus, it is not aggregation *per se* that represents a problem, but rather the fact that tight aggregates cannot be dispersed without damaging the cells. The situation is analogous to that of balloons taped together: the tape itself does not break the balloons, but trying to separate the balloons will cause them to break. These considerations apply not only to aggregated cells, but also to cells attached to a plastic surface via surface-bound Abs (which would be analogous to balloons taped to a wall), as has been used with anti-CD20 Abs (Mathas *et al*, 2000; Cragg and Glennie, 2004). Chan *et al* (2003) reported that certain Abs to CD20 were able to induce apoptosis and that the ability to induce apoptosis was correlated with the extent of aggregation induced by the Ab; this suggests the possibility that it was the aggregation itself that was responsible for some of the effects. One approach to circumvent the effect of aggregation would be to grow cells in soft agarose, which would prevent cell aggregation but would allow Ab binding.

A sixth problem with apoptosis assays is the frequent lack of microscopic observation: many types of errors would be readily revealed in this way. FACS data alone allow many errors in interpretation, such as the counting of subcellular fragments as apoptotic cells. In conclusion, these data demonstrate that some widely used assays for apoptosis do not actually measure apoptosis, but instead detect any type of cell death, apoptotic, or necrotic. This statement is not meant to imply that apoptosis is not an important mechanism of death in many situations, or that reliable assays for apoptosis are not available. Annexin V–PI binding is reliable if the size of the apoptotic cells is described. Measurement of cleaved caspase 3 or PARP is reliable if specificity controls are adequate. The modified JC-1 assay, combined with Sytox green, appears to be reliable. The PI assay for sub-G_1_ DNA is not reliable and should not be used. These considerations will be helpful in understanding the mechanisms by which tumours cells are killed.

## Figures and Tables

**Table 1:** Synopsis of problems with assays used to detect apoptosis

**Method**	**References^a^**	**Potential problem**
PI staining	(Shan *et al*, 1998; Chan *et al*, 2003; Jazirehi *et al*, 2003)	Does not distinguish between necrotic and apoptotic cells.
Staining for mitochondrial membrane potential	(Darzynkiewicz *et al*, 2001; Chan *et al*, 2003; Jazirehi *et al*, 2003; Carlo-Stella *et al*, 2006)	Does not distinguish between necrotic and apoptotic cells (unless JC-1 is combined with Sytox green).
Ab detection of cleaved PARP, caspase 3, or other markers	(Dai and Krantz, 1999; Darzynkiewicz *et al*, 2001; Jazirehi *et al*, 2003)	Nonspecific staining may be increased. Must include negative control Ab.
Annexin V staining	(Shan *et al*, 2000; Darzynkiewicz *et al*, 2001; Cragg and Glennie, 2004)	Subcellular fragments may obscure results. Must monitor size of objects counted in the FACS.^b^

Abbreviations: Ab, antibody; FACS, fluorescence-activated cell sorter; PARP, poly(ADP-ribose) polymerase; PI, propidium iodide.

a References cited are examples in which these methods were used, chosen from a large number of such publications.

b This problem applies to all assays which are scored with the FACS.
